# Is Micronutrient Supplementation Helpful in Supporting the Immune System during Prolonged, High-Intensity Physical Training?

**DOI:** 10.3390/nu16173008

**Published:** 2024-09-05

**Authors:** Francesca Felice, Roberta Moschini, Mario Cappiello, Gemma Sardelli, Rossella Mosca, Lucia Piazza, Francesco Balestri

**Affiliations:** 1 Biochemistry Unit, University of Pisa, Via San Zeno 51, 56123 Pisa, Italy; francesca.felice@unipi.it (F.F.); roberta.moschini@unipi.it (R.M.); gemma.sardelli@phd.unipi.it (G.S.); r.mosca@studenti.unipi.it (R.M.); lucia.piazza@phd.unipi.it (L.P.); francesco.balestri@unipi.it (F.B.); 2 Interdepartmental Research Center Nutrafood “Nutraceuticals and Food for Health”, University of Pisa, 56123 Pisa, Italy

**Keywords:** micronutrients, intensive exercise, immune susceptibility, nutrition, physical activity, sports nutrition

## Abstract

It is well known that during prolonged, high-intensity physical training, athletes experience a state of immunosuppression and that balanced nutrition can help maintain immunity. This review summarizes the effects (amplified by virus infection) of high-intensity, long-term exercise on immunity, critically presenting key micronutrients and supplementation strategies that can influence athletes’ performance and their immune system. The main conclusion is that micronutrient supplementation with diet could help to protect the immune system from the stress effects induced by intense physical activities. The importance of personalized supplementation has been also recommended.

## 1. Introduction

The immune system is important for protecting against diseases, including all infectious agents. Studies on the relationship between immunology and exercise have evidenced that moderate exercise appears to exert a protective effect on the immune system, while repeated bouts of exhaustive exercise can result in immune dysfunction [1]. Recently, exercise immunology studies have been extensively revised [2], showing that the nature, intensity, and duration of physical exercise can positively or negatively affect the immune system. This debated aspect of sports training depends on multifactorial factors, with factors such as the neuroendocrine, metabolic, or nutritional status of athletes among the most important. Certainly, during aerobic exercise, exposure of the lungs to airborne viruses increases because of the higher rate and depth of breathing [3,4]. Moreover, high-level athletes are very frequently exposed to exhausting training programs, sleep disturbances, travel, and psycho-social and environmental stressors [5,6]. All these are potential immune dysregulatory factors, increasing the likelihood of illness.

In order to minimize these phenomena and to improve recovery, nutritional interventions are often recommended to athletes due to the fact that they are able to exert a role in the immune and inflammatory changes following vigorous exercise, as reported by the consensus statement [7]. 

The first effective ratification on the role of nutrients in sports activity was indicated in a document drawn up in 2019 by members of the Commission of Experts of the Union of European Football Associations (UEFA) correctly stating that “An evidence-based approach to nutrition emphasising, a ‘food first’ philosophy (i.e., food over supplements), is fundamental to ensure effective player support” [8].

Athletes, particularly those competing in endurance events, often experience a high incidence of illnesses after intense physical exercise, especially respiratory tract infections. In this review, we discuss the effects of an intensive exercise training program, which can be amplified by virus infections, such as Severe Acute Respiratory Syndrome Coronavirus 2 (SARS-CoV2), on immune functions in athletes. In recent years, there has been growing interest in analyzing the causes, symptoms, and frequency of these conditions, as well as in exploring ways to alleviate them through supplements, sports nutrition, or immunonutrition strategies. We also analyze some key micronutrients, such as vitamins C, D, and B, magnesium, and selenium, known as immune modulators, and provide a critical view of the actual effectiveness of their use as dietary supplements.

## 2. The “J-Shaped Curve” and the “Open Window” Theory

**Figure 1:**
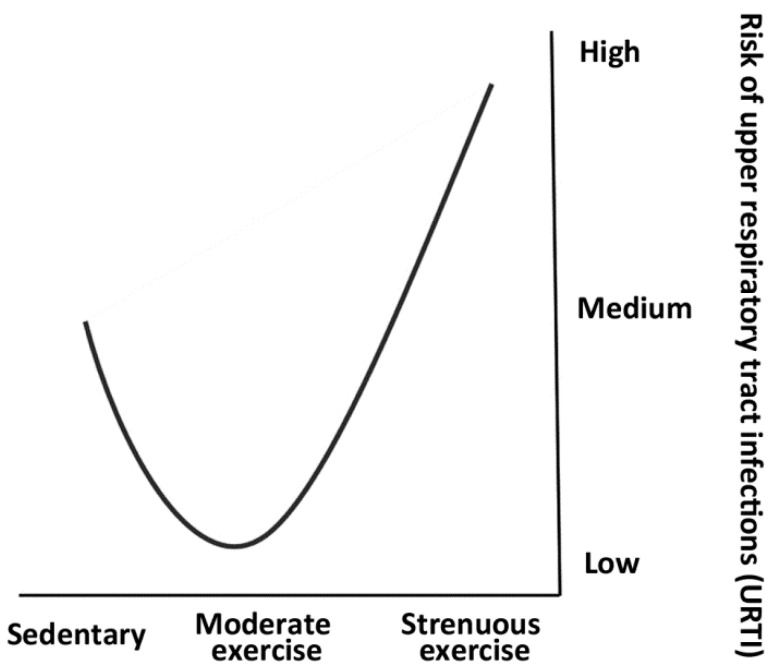
Risk of illness in response to exercise: “J-shaped Curve”. Strenuous endurance exercise (>70–75% VO_2_max) increases the risk of illness; conversely, moderate physical activity decreases the risk of illness compared with sedentary individuals [9].

It has been observed that extended exercise sessions and intense training are associated with a higher risk of infections, including respiratory tract infections, and their related symptoms. The risk of infections in response to exercise has been modeled as a “J-shaped Curve” (Figure 1) [9]. According to this model, the risk of acquiring a respiratory tract infection is lower in people who engage in moderate physical activity compared to sedentary individuals. On the contrary, an acute bout of prolonged (>2 h) and vigorous aerobic exercise (>6 metabolic equivalents (MET), i.e., the amount of oxygen consumed while sitting at rest, equal to 3.5 mL O_2_ per kg body weight × min) may suppress immune function, thus increasing the risk of illness [10,11]. Recently, it has been suggested that regular bouts of short-duration (i.e., up to 45 min), moderate-intensity exercise are ‘immuno-enhancing’ [12,13], whereas repeated bouts of long-duration (>2 h), high-intensity exercise can be ‘immunosuppressive’ [14]. To explain the reason for the J-curve, research has focused on the role of the immune system: after prolonged and high-intensity exercise (>2 h), there is a period, called the “open window”, of post-exercise immunosuppression with variable duration, between 3 and 72 h, depending on the parameters taken into consideration, such as cortisol levels, redox status, and white blood cell count or change in proinflammatory cytokine levels, catecholamines, and prostaglandins released in response to the exercise session [15,16].

However, the question of whether athletes are more susceptible to illness/infection than the general population continues to be discussed. Simpson et al. [17] proposed a debate article among researchers supporting opposite theories on the affection of the immune system by exercise, increasing susceptibility to infections. The former states that the presence of stressors, such as physiological, metabolic, genetic, psychological, or infection/allergenic exposure, can promote immune dysfunction. The latter claims that the detrimental effects of exercise on immunity are inappreciable and/or research has not been designed optimally to assess immune competency. All of the experts, however, agree that infection susceptibility has a multifactorial background. Even this recent debate concluded that the issue remains unsolved and needs more empirical research on exercise immunology [18].

## 3. What Happens to Immune Defense after Prolonged, High-Intensity Exercise in Athletes?

As reported above, when the bout of exercise is prolonged and at high intensity (more than 20 h, 15 METs, per week), a transient depression of the innate immune system occurs in athletes [2,4,18,19,20,21,22]. There is a significant increase in lymphocyte and neutrophil levels but a decrease in phagocytic activity, a reduction in white blood cell levels, and a reduced oxidative burst of neutrophils after exercise, which can last for many hours [18,22]. Furthermore, after exercise, a decline in the concentration of all lymphocyte subpopulations has been observed; in particular, the number of circulating natural killer cells (NK-cells) decreases to less than one-half of normal levels and is reestablished within 24 h [23]. If the bout of exercise is prolonged (>2 h) and strenuous (>6 METs), the reduction in NK-cell counts and cytolytic activity may start in the course of the exercise session [23]. The number of circulating lymphocytes decreases below pre-exercise levels and remains at low levels for several hours after exercise [24]. This exercise-induced lymphopenia mainly concerns the lymphocyte subtypes with potent effector functions out of the blood, such as NK-cells and CD4+ T helper (Th) and CD8+ T cytotoxic (Tc) cell phenotypes [4]. Prolonged strenuous exercise decreases the percentage of circulating type 1 T lymphocytes (both Th1 and Tc1), which are known to secrete interferon-γ, IL-2, and TNF-α [20]. 

However, controversial results regarding NK-cell count and cytolytic activity are present, due to strongly varying methods used to detect NK-cell cytotoxicity, as evidenced by the works of Zimmer and co-authors [25,26]. Thus, further research in this field is necessary.

The decrease in both the number and percentage of T cells after exhaustive exercise and the critical role played by type 1 T cells in the generation of antiviral defense could be the origin of vulnerability to various viral infections [27] as recently observed for COVID-19 infection [28,29]. 

Strenuous exercise induces a systemic inflammatory response followed by a plasma increase of substances that are known to influence lymphocyte functions, such as pro- and anti-inflammatory cytokines such as IL-1, IL-6, IL-10, and IL-1 receptor antagonist (IL-1ra), with a parallel decrease in the percentage of circulating T cells [30]. Among inflammatory mediators activated by intensive exercise, the main one is IL-1β which is able to regulate a broad range of immune responses and participate in several physiological processes. The production of IL-1β requires the presence of the NLRP3 inflammasome, but the intensity of aerobic exercise activity produces different responses, since after chronic exercise at high intensity, a significant increase in the expression of the NLRP3 gene and serum IL-1β and IL-18 levels was observed, while acute aerobic exercise at moderate intensity showed no effect [31].

Some studies have also assessed the impact of prolonged training on mucosal immunity [32,33,34]. Respiratory viruses are infectious mediators that initially invade respiratory mucosal tissue, replicating within the host’s living cells. Salivary (s-IgA) and mucosal immunoglobulins recognize and bind viral epitopes, blocking their entry into mucosal cells. Experimental data evidenced a significant reduction of inactive serum and s-IgA levels in athletes following prolonged and strenuous training with a parallel increase in the incidence of viral respiratory infections. 

During sports activities, airborne and droplet-borne pathogens, such as common cold viruses, are easily transmitted by aerosol or contact [35]. If the virus can enter the airways, through a debilitated immune system in which the defensive barrier of immunoglobulins is impaired and the ability to remove virus-infected cells is deficient, the virus is more likely to replicate, developing into a symptomatic infection. Moreover, if resting periods between the exercise sessions/competitions are not long enough to allow immune function to recover, increased sensitivity to infectious diseases may be present for a prolonged period of time [36].

## 4. Nutraceutical Strategies for Restoring Immunity after Exercise

While there is no specific method that completely removes the risk of contracting any infection, there are numerous approaches to “counterattack” the “open window” phase. If it is not possible to sustain athletes with adequate nutritional proposals, one possibility is to give appropriate immune-nutritional support such as micronutrients, reducing the effect of exercise-induced immunodepression. As detailed below, minerals such as zinc or magnesium and vitamins such as C, D, and B are crucial for maintaining a healthy immune system and preventing exercise-induced immunodepression. Zinc is essential for immune cell function by helping in the production of antibodies, while magnesium supports immune response by regulating inflammation and muscle function. Vitamins, such as C and D, act as antioxidants, reducing oxidative stress caused by intense physical activity [37]. Together, these micronutrients contribute to maintaining a balanced immune system, reducing the risk of infections and illness that can occur after strenuous exercise. However, a relevant effect of exercise and training is the reduction in micronutrient concentration and availability.

## 5. Micronutrients

A general and relevant role of micronutrients is to support the function of the immune system at every stage of life [38] and play a key role in fighting free radicals. Clinical deficiencies of micronutrients are known to unfavorably affect the immune system, predisposing people to infections. Micronutrient deficiencies, which are ubiquitous even in industrialized countries [39], favor the risk of morbidity and mortality associated with measles, diarrheal disease, pneumonia [40,41,42], and all common infections [43]. There is limited information on immune system response when micronutrient levels are not present in optimal amounts but that are not yet so scarce as to cause symptoms of a real deficiency. However, infections and malnutrition are closely linked; in fact, the immune response to an infection can worsen nutritional deficiencies and increase the body’s need for micronutrients [44]. 

The recommended daily allowance (RDA) for all nutrients is known, which is the average daily intake level required to avoid clinical or subclinical deficiencies in the mainstream (97–98%) of a healthy general population. Many people have an insufficient daily intake of micronutrients, and if we consider that intense physical exercise induces a great loss in their quantities, the main conclusion is that nutrients should be integrated [45,46,47].

The primary mission in nutritional immunology is to give an indication of the necessary levels of micronutrients needed to preserve immunological balance after prolonged and intensive exercise [48,49]. Prolonged and intensive physical training requires higher levels of micronutrients than for individuals with sedentary habits because of the modulating effects of sweat and urine losses on mineral metabolism [47]. The importance of micronutrient supplementation has been expressed recently by a consensus statement [7]. Various nutritional agents are known for their ability to reduce the risk of infection and attenuate immune changes and inflammation that can occur after intensive exercise [49]. We focus on vitamins C, D, and B, for which there is the strongest evidence, and on zinc, magnesium, and selenium, which are known to exert modulatory effects on immune system function.

### 5.1. Vitamin C

Vitamin C is generally recognized for its antioxidant and immunomodulating effects [50,51]. Humans cannot synthesize vitamin C, which makes it an essential nutrient. The RDA for vitamin C is 90 mg/day for adult men and 75 mg/day for adult women to maintain a near-maximal neutrophil concentration with minimal urinary excretion of ascorbate [52]. The level of vitamin C intake that is sufficient to meet the needs of almost all (97.5%) healthy subjects in a specific population group (PRI, Population Reference Intake) is 105 and 85 mg/day for adult men and women, respectively [53].

The content of vitamin C in immune cells ranges from 3.5 to 1.5 μΜ, depending on the cellular type considered. The intracellular percentage of vitamin C is important because it is a cofactor of numerous enzymes involved in the metabolism and epigenetic control of immune cells [51].

Vitamin C, stimulating neutrophil migration, plays a key role as barrier infection, stimulating neutrophil migration into the site of infection. Vitamin C is involved in the regulation of proliferation, differentiation, and activity of immune cells, including macrophages and T lymphocytes; it enhances the activity of both natural killer lymphocytes and macrophages [54]. During an infection, vitamin C levels decrease by 50% since inflammatory cytokines negatively regulate an isoform of the sodium-dependent vitamin C cellular transporter [55]. A meta-analysis [56] has shown that higher vitamin C supplementation than the RDA (100–200 mg/day) is useful in alleviating the severity and duration of the common cold, which is athletes’ major disease during transient immunodepression in the early post-exercise period. Another meta-analysis [57] evidences the beneficial effect of vitamin C supplementation in athletes practicing intensive exercise, in which 0.5 to 2 g/day of vitamin C prevented exercise-induced bronchoconstriction [58]. Moreover, it has been observed that vitamin C administration contributes to decreasing the risk of developing more serious respiratory infections, such as pneumonia [59]. However, reaching adequate levels (100–200 mg/day) after infection or to decrease the risk of infection can be difficult, as the RDA is often not even met [52]. At present, for vitamins, it is safe to recommend that athletes keep the five fruit and vegetable servings (corresponding to 200 mg/day) in order to consume an adequate amount of vitamin C, while higher doses of vitamin C are not recommended [60].

### 5.2. Vitamin D

Over the past decade, the role of vitamin D has become much more recognized in regulating immune system cells thanks to the discovery of the vitamin D receptor (VDR) and essential vitamin D-metabolizing enzymes found in immune cells. Regulation of the immune system depends on the concentration of vitamin D; the vitamin D receptor is expressed in all immune cells, which are also able to convert 25-hydroxyvitamin D3 (25(OH)D3) into the active form 1,25-dihydroxyvitamin D3 (1,25(OH)2D3), which is responsible for immuno-regulatory activity [61]. A low percentage of vitamin D is introduced through diet (20%), while the rest is produced upon exposure to solar radiation (especially UVB). The active form, 1,25(OH)2D3, stimulates the synthesis of two families of antibacterial peptides (cathelicidin and defensin), synthesized by macrophages, monocytes, and other cells of the innate immunity, as well as epithelial cells lining the respiratory tract [62]. Vitamin D can stimulate the innate immune system not only in activating TLR receptors or increasing cathelicidin and β-defensin expression but also in modulating T cell function and the adaptive immune response both by decreasing immunoglobulin secretion from plasma cells and modulating the production of pro-inflammatory cytokines [63].

The US Institute of Medicine [64] and the European Food Safety Authority [65] recommend a tolerable upper intake of vitamin D of 4000 IU/day. However, especially in Europe, vitamin D intake (as well as vitamin E, folate, and selenium) does not reach recommended doses. Thus, it is improbable that the RDA for vitamin D will increase the serum levels necessary for appropriate immune system function [66]. 

Five meta-analyses have shown that vitamin D (300–3653 IU/day) in adults and children can decrease the risk of respiratory tract infection [67], particularly in subjects with hypovitaminosis D [68].

Monitoring vitamin D status is important in athletes since diminished immune function and increased risk of respiratory infection are seen in individuals with low vitamin D status. Recent evidence suggests that athletes exhibiting “sufficient” vitamin D concentrations (>50 nmol/L) may still be at an increased risk of contracting a respiratory tract infection compared with athletes with a concentration higher than 75 nmol/L [69]. As reported by Bermon et al. [13], elite athletes manifest vitamin D deficiencies, even those living in sunny climates and who train in outdoor environments [70,71], likely due to the use of high-factor sunscreen creams. The studies highlight the preventive role of vitamin D supplementation in respiratory tract infections. The best advice is to monitor the athlete’s vitamin D concentration by focusing on the increase in the target concentration > 75 nmol/L (1000 IU).

### 5.3. Vitamins B

B-complex vitamins (thiamin, riboflavin, vitamin B-6, B-12, and folate) are a group of water-soluble vitamins that play an active role in humoral and cell-mediated immunity by acting as coenzymes in many catabolic and anabolic enzymatic reactions. Vitamin B deficiency has been linked to various types of disorders. Vitamins B-6 and B-12 belong to the B-complex vitamins that play a primary role in immune function since their deficiency causes a reduction in the production of IL-2, lymphocytopenia, etc. [72]. Furthermore, their deficiency has suppressive effects on NK-cell activity [73]. Even in developed countries, in the absence of an optimal diet, a significant percentage of the population suffers from deficiencies or insufficiencies of B-complex vitamins. Concerning vitamin B-6, a study showed that its supplementation in young women at doses up to 2.1 mg/day (higher than the RDA of 1.3 mg/day) for one week improved the proliferation of lymphocytes in a dose-dependent manner [74].

Due to the involvement of B-complex vitamins in maintaining innate immunity and NK-cell function, the integration of the entire group of B-vitamins, rather than one, would be a rational approach for preserving general health [75,76], particularly in athletes performing prolonged and exhaustive exercise [77]. B-complex vitamins play an important role in maintaining the health of the active individual, ensuring that energy can be produced for physical activity. As reported by Woolf et al. [77], dietary mean intakes in athletes, especially in women, do not reach recommended levels. Athletes who have poor or marginal nutritional status for a B-complex vitamin may have a decreased ability to perform exercise at high intensities [78]. The International Society of Sports Nutrition (ISSN) has shown the ergogenic benefit of B-complex vitamins, recommending the consumption, in well-nourished athletes, of the entire complex of B vitamins according to the RDA [79]. 

Recently, the involvement of vitamin B complex has also been shown in cytokine storm. For example, it was observed that vitamin B-6 (as well as B-2 and B-9) upregulated IL-10, a powerful anti-inflammatory and immunosuppressive cytokine, and inhibited antigen-presenting cells and T cells [80]. Vitamin B-complex not only helps to build and maintain a healthy immune system but could also potentially prevent or reduce viral respiratory symptoms. It may be wise to recommend the use of vitamin B complex in athletes performing prolonged and exhaustive exercise, in accordance with the ISSN recommendation [79].

### 5.4. Selenium

Selenium plays a key role in the immune system and its concentration below 1400 µg/L in human serum is non-toxic [81]. The immune system needs an adequate daily intake of selenium, whose bioavailability depends on numerous factors including its form (the organic one is the most bioavailable) [82] and its conversion into metabolites which is mainly dependent on individual genetic elements [83]. Selenium is important for the function of seleno-proteins that act like redox regulators [38]. Selenium deficiency causes a decline in immune response through reduced NK-cell cytotoxicity, defective humoral and cell-mediated immunity, reduced plasmatic immunoglobulin titers, increased viral virulence, and diminished response to vaccination [42]. On the contrary, the impact of its supplementation (50 µg/daily) increases cell-mediated immunity, proliferation and differentiation of T helper cells, and immune response to viruses in selenium-deficient subjects [83]. 

Selenium deficiency, although rare, can be evaluated by measuring serum selenium concentrations as well as plasma or serum glutathione peroxidase 3 (GPx-3) activity or seleno-protein P levels [84]. It is important to evaluate selenium levels in athletes because its metabolism in the body can change during exercise. A negative correlation, indeed, was found between pre- and post-exercise selenium levels, related to redox status, GPx levels, and the heart morphology of athletes [85]. 

Selenium has not been classified as an immunonutrient for exercise in the consensus due to the lack of scientific evidence on the potential boost to the immune system or the prevention of exercise-induced immunodepression through supplementing non-deficient athletes with selenium [7]. 

However, it has been demonstrated that integration with sodium selenite, unlike selenate, can render viruses unable to penetrate the healthy cell membrane, oxidizing the thiol groups in viral protein disulfide isomerase, thus abolishing their infectivity [86]. In light of the reported data, supplementation in the regular diet is necessary only in selenium-deficient athletes.

### 5.5. Magnesium

Magnesium (Mg) is an essential element that plays an important role in the control of maintaining enzyme activity, membrane function and integrity, and cell signal transduction. In the immune system, Mg is involved in different processes such as immunoglobulin synthesis, activation of macrophages, and immune cell adherence [7].

Mg serum concentration depends on the intensity and duration of exercise since an increase in extracellular Mg was found in athletes who underwent short-term (<1.5 h), high-intensity exercise, while, on the contrary, athletes who performed prolonged and intensive exercise showed hypomagnesemia [87]. 

Depletion of Mg was observed after intensive training, suggesting that its supplementation (300–500 mg/day for 1–4 weeks) can improve muscle performance (such as fatigue resistance) and reduce exercised-induced inflammation, DNA damage, cortisol plasma level, and immunological blood markers [7,88]. Mg supplementation of 500 mg/day is recommended in athletes where there is a measured deficiency. Currently, there are no studies available on higher supplementation of Mg and the effects it may induce. More research needs to be carried out on longer supplementation in athletes.

### 5.6. Zinc

Innate or non-specific immunity is altered by suboptimal plasmatic zinc concentrations. Correction of zinc deficiency through its supplementation reduces mortality from infectious diseases [49]. Zinc also plays an essential role in cell-mediated and humoral immunity [89]. Moreover, it can modulate the release of cytokines and the induction of CD8 T cell proliferation which are involved in defense against pathogens [90]. Its efficacy depends on the type of zinc salt used, with zinc acetate offering greater benefits than other zinc salts [91]. 

Exercise has a pronounced effect on zinc metabolism [92], since athletes have a higher loss of this metal in their sweat and urine than sedentary individuals. An imbalanced diet might be the primary cause of the zinc deficiencies often observed in athletes [93]. The efficacy of zinc supplementation as a treatment for the common cold has been investigated in numerous studies [94], revealing that its intake should take place within 24 h of the onset of symptoms to be of any benefit [95]. 

The accepted RDA of zinc is 8–11 mg/day for adults, but it has been proposed that a zinc intake of 30–50 mg/day may help fight against viral infection [96]. However, according to Walsh [97], high-dose zinc supplementation can decrease immune function and should be avoided. Thus, supplementation of zinc at 30 mg/day for optimal immune function in athletes is recommended. 

In Table 1, we reported micronutrient supplementation in high-level athletes.

## 6. Discussion 

An efficient immune system, deficiencies in which can be observed after prolonged and intense physical exercise, is fundamental to fighting against infections and their consequences. For this reason, the sports medicine community needs to establish the best conditions for improving immunity in athletes who, as debated, are at a higher risk of infections. Thus, personalized diets and supplement strategies are gaining more and more relevance. 

It is generally argued that the use of supplementation with micronutrients can represent a real benefit mainly for athletes, continuously engaged in prolonged and intense training programs. If their dietary ration is limited in energy intake (lower than “energy availability” recommended) for the adoption of extreme weight loss practices or for the elimination of one or more food groups from their diet that reduce optimal micronutrient intake, supplementation is of particular importance.

Clear deficiencies of nutrients required for proper immune function must be uncovered and corrected. Many athletes, especially at a young age, do not appreciate the importance of diet for the maintenance of a healthy immune response and for recovery from injury and illness, but nutritional strategies for enhancing immunity need to be strongly recommended.

Taking care of the immune system using dietary supplements might be helpful to improve the natural defenses of the athlete’s body, particularly in persons who are more sensitive to the “open window”, such as those whose discipline requires vigorous bouts of exercise or close contact. 

Among trace elements, selenium and zinc have shown the most favorable immune-modulatory effects in viral respiratory infections. However, although selenium may reduce oxidative stress during aerobic exercise [79], improvements in aerobic capacity have not been demonstrated in supplemented, non-deficient athletes. 

Athletes must be screened for malnutrition and micronutrient deficiencies using validated nutrition screening tools (e.g., Nutrition Risk Score-2002) [98], particularly those who have suffered respiratory diseases. Nutritional strategies that can support the immune system have the potential to enhance defense against viral infections, protecting athletes from viral respiratory illnesses.

## 7. Conclusions 

The immune system requires the support of many nutrients, and micronutrient supplementation in the diet could help in its protection against the stress effects induced by intense physical activities. A carefully personalized athletes’ anamnesis, considering the peculiarities of different sports (high-intensity, interval training, or exhausting training programs) and all of the non-exercise immune stressors (sleep disturbances, travel, and psychosocial and environmental stressors), should be recommended. Moreover, a personalized athlete approach to selected immune micronutrients that reflects their bioavailability, effective doses, body concentrations, and synergistic effect might be appropriate. Finally, it is fundamental to set a direction for more targeted research efforts in the future.

## Figures and Tables

**Table 1:** Micronutrient supplementation in high-level athletes.

	RDA *	Regular Diet (Dose Suggested)	Supplement (Dose Suggested)
Vitamin C	Males 90 mg/d Females 75 mg/d	200 mg/d	-
Vitamin D	200 IU/d (age < 51)	-	1000 IU/d
B-complex vitamins:		as RDA	-
Thiamin (B-1)	Males 1.2 mg/d Females 1.1 mg/d		
Riboflavin (B-2)	Males 1.3 mg/d Females 1.7 mg/d		
B-6	1.3 mg/d (age < 51)		
B-12	2.4 µg/d		
Folate	400 µg/d		
Selenium	55 µg/mL	50 µg/mL	-
Zinc	Males 11 mg/d Females 8 mg/d	-	30 mg/d
Magnesium	Males 420 mg/d Females 320 mg/d	-	500 mg/d

* Recommended dietary allowances (RDA) based on the 2002 Food and Nutrition Board, National Academy of Sciences–National Research Council recommendations.
